# Glaucocalyxin A Inhibits Platelet Activation and Thrombus Formation Preferentially via GPVI Signaling Pathway

**DOI:** 10.1371/journal.pone.0085120

**Published:** 2013-12-30

**Authors:** Wei Li, Xiaorong Tang, Wenxiu Yi, Qiang Li, Lijie Ren, Xiaohui Liu, Chunjun Chu, Yukio Ozaki, Jian Zhang, Li Zhu

**Affiliations:** 1 Cyrus Tang Hematology Center, Soochow University, Suzhou, China; 2 College of Pharmaceutical Science, Soochow University, Suzhou, China; 3 Department of Laboratory Medicine, Faculty of Medicine, University of Yamanashi, Chuo, Yamanashi, Japan; King’s College London School of Medicine, United Kingdom

## Abstract

Platelets play a pivotal role in atherothrombosis and the antiplatelet agents have been proved to be useful in preventing onset of acute clinical events including myocardial infarction and stroke. Increasing number of natural compounds has been identified to be potential antiplatelet agents. Here we report the antiplatelet effect of glaucocalyxin A (GLA), an *ent*-diterpenoid that we isolated and purified from the aerial parts of *Rabdosia japonica* (Burm. f.) var. *glaucocalyx* (Maxim.) Hara, and investigate the molecular mechanisms by which GLA inhibits platelet activation and thrombus formation. The effect of GLA on platelet activation was measured using platelets freshly isolated from peripheral blood of healthy donors. Results showed that pretreatment of human platelets with lower concentrations of GLA (0.01μg/ml, 0.1μg/ml) significantly inhibited platelet aggregation induced by collagen (P<0.001) and CRP (P<0.01), a synthetic GPVI ligand, but not by ADP and U46619. Accordingly, GLA inhibited collagen-stimulated tyrosine phosphorylation of Syk, LAT, and phospholipase Cγ2, the signaling events in collagen receptor GPⅥ pathway. GLA also inhibited platelet p-selectin secretion and integrin activation by convulxin, a GPVI selective ligand. Additionally, GLA was found to inhibit low-dose thrombin-induced platelet activation. Using a flow chamber device, GLA was found to attenuate platelet adhesion on collagen surfaces in high shear condition. In vivo studies showed that GLA administration increased the time for complete occlusion upon vascular injury in mice, but did not extend tail-bleeding time when mice were administered with relatively lower doses of GLA. Therefore, the present results provide the molecular basis for the inhibition effect of GLA on platelet activation and its in vivo effect on thrombus formation, suggesting that GLA could potentially be developed as an antiplatelet and antithrombotic agent.

## Introduction

Once vascular injury has occurred, platelets are principally activated by locally exposed collagen in the vascular wall and locally generated thrombin, initiating hemostasis[1]. The binding of collagen to GPVI on platelets results in receptor clustering and thereby stimulates the tyrosine phosphorylation of specific tyrosine residues within an associated trans-membrane protein, the Fc receptor γ-chain (FcRγ-chain)[2,3]. This leads to the recruitment of signaling proteins such as the Src kinase, the tyrosine kinase Syk, PLCγ2, phosphoinositide 3-kinase (PI3K) and MAPKS[3,4], resulting in the inside-out activation of the integrin αIIbβ3 and the release of the secondary mediators, such as ADP and thromboxane A2 (TxA2), culminating in platelet aggregation mediated by fibrinogen binding to αIIbβ3 and thrombus formation. 

The modulation of platelet activity using specific pharmacological agents has proven to be a successful strategy for the prevention of thrombosis[5]. Mechanistically, current antiplatelet drugs include ADP antagonists, COX-1 inhibitors, antagonists of the major platelet integrin αIIbβ3, and phosphodiesterase inhibitors. However, the risk of uncontrolled bleeding due to their inherent antihemostatic effects limited their clinical use[6]. Therefore, tremendous effort has been made in the past years on the identification of novel pharmacological targets with both effective and safe antiplatelet effect to prevent occlusive thrombus formation in myocardial infarction and stroke. The search for compounds to prevent platelet activation has included the investigation of natural compounds that are able to inhibit platelet function, such as quercetin[7], polyphenols[8], and salvianolic acid A[9]. 

*Rabdosia japonica* (Burm. f.) var. *glaucocalyx* (Maxim.) Hara is a perennial herb that is distributed widely in East Asia, and the dried whole plant of *Rabdosia japonica* (Burm. f.) var. *glaucocalyx* (Maxim.) Hara has been used traditionally as a folk medicine for treating gastrointestinal disorders, tumors, and inflammatory diseases[10,11]. Recent reports showed that glaucocalyxin A (GLA) isolated from *Rabdosia japonica* has an anti-neuroinflammatory effect on LPS-stimulated microglial cells[12], strong cytotoxic effects on normal liver cell line BRL and several tumor cell lines in vitro[13], and apoptotic effects on human leukemia HL-60 cells through mitochondria-mediated death pathway or GSH perturbation[14,15]. Although there were studies of GLA effect on rabbit platelet function[16,17], the mechanism by which GLA affects platelets and its effect on thrombus formation in vivo remains unclear. In the present study, we tested the effect of GLA on platelet activation in response to a variety of agonists and thrombus formation in vivo. We found, for the first time, that lower doses of GLA inhibits collagen- and thrombin-induced platelet activation and decreases thrombus formation without bleeding tendency.

## Materials and Methods

### Animals and human samples

All animal procedures were approved by the University Committee on Animal Care of Soochow University. Human venous blood was obtained from healthy donors in accordance with the Declaration of Helsinki and the permission from the University ethical committee of Soochow University. All participants gave written informed consent.

### Materials

Thrombin, ADP, HEPES and bovine serum albumin (BSA) were purchased from Sigma (St Louis, MO, USA). Collagen was purchased from Chrono-Log Corp (Havertown, PA, USA) and U46619 was from Calbiochem (Germany). Convulxin was purchased from Alexis Biochmicals (Alx-350-100-C050, USA) and CRP was synthesized in Peptide Institute (Osaka, Japan). Antibodies to Syk and phospho-Syk, LAT and phospho-LAT, and PLCγ2 and phospho-PLCγ2, were from Cell Signaling Technology (Beverly, MA, USA). 

### Extraction and Isolation of GLA

**Figure 1:**
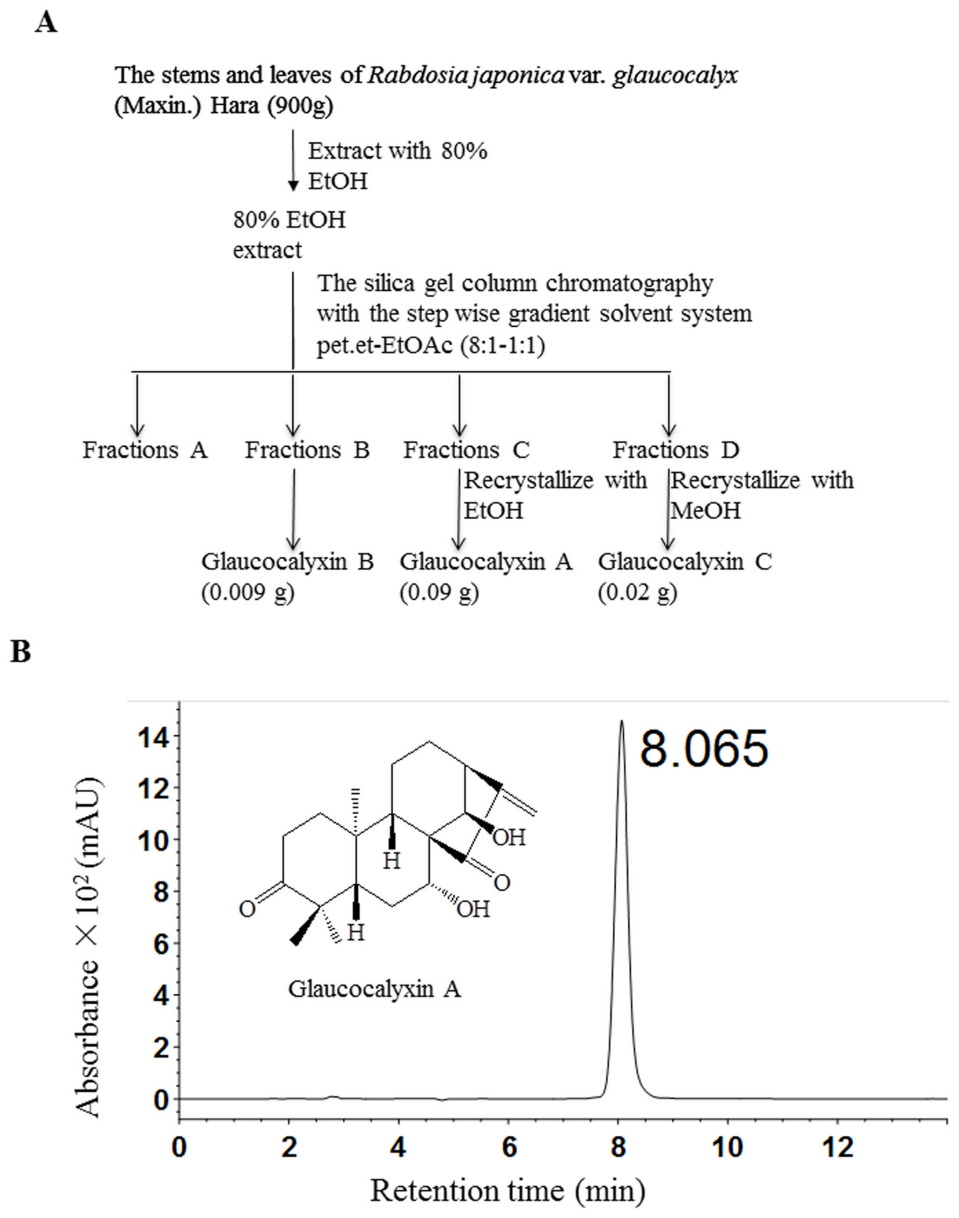
Isolation and identification of Glaucocalyxin A. *Rabdosia japonica* (Burm. f.) var. *glaucocalyx* (Maxim.) Hara were chopped and extracted, and the extract was applied to column chromatography. GLA, GLB, and GLC were obtained from the fraction C, fraction B, and fraction D, respectively (A). The isolated GLA was further confirmed by HPLC and its purity is 98% (B).

Stems and leaves of *Rabdosia japonica* (Burm. f.) var. *glaucocalyx* (Maxim.) Hara were collected in Tieli of Heilongjiang Province in September 2009 and air-dried, and no permission is required to collect the stems and leaves of Rabdosia Japonica. The identity of the plant material was verified by Prof. Zhenyue Wang, College of Pharmacy, Heilongjiang University of Chinese Medicine, Haerbin, China. A voucher specimen（altitude 280.4m; N 46°986'; E128°688'）is deposited in the Herbarium of Heilongjiang University of Chinese Medicine, Haerbin, China. As shown in Figure 1A, the dried and powdered stems and leaves (900g) were chopped and extracted with 80% EtOH two times under reflux and concentrated under vacuum to yield an EtOH extract. The extract (74.0g) was applied to column chromatography over a silica gel (200-300 mesh, 1200g) column eluted with a system of petroleum ether:ethyl acetate (pet.et:EtOAc) (8:1; 4:1; 2:1; 1:1) to obtain fractions A-D. GLA (0.09g) was obtained by recrystallization in EtOH from the fraction C (10.0g). GLB (0.009g) was from the fraction B (8.0g) and GLC (0.02g) was obtained by recrystallization in MeOH from the fraction D (42.0g). 

The structure of GLA, GLB, and GLC were determined by spectroscopic analyses, including MS and 1D-NMR spectra. GLA: colorless square crystal (EtOH), APIMS m/z: 355.2 [M+Na]^+^. ^1^H NMR (400MHz, DMSO-*d*_6_) δ: 5.95 and 5.40 (each 1H, brs, 17-H_a/b_), 4.71 (1H, brs, H-14α), 4.03 (1H, m, 7β-H), 2.95 (1H, brs, 13α-H), 1.04 (3H, s), 1.00(3H, s), 0.98 (3H, s). ^13^C NMR (100MHz, DMSO-*d*_6_) δ: 37.9 (1-C), 33.9 (2-C), 216.5 (3-C), 46.5 (4-C), 50.6 (5-C), 30.9 (6-C), 73.0 (7-C), 60.7 (8-C), 52.8 (9-C), 38.6 (10-C), 18.1 (11-C), 30.3 (12-C), 46.1 (13-C), 74.6 (14-C), 207.0 (15-C), 148.8 (16-C), 116.9 (17-C), 27.4 (18-C), 21.1 (19-C), 18.1 (20-C). GLA was further analyzed by HPLC for its purity. GLA was dissolved in methanol at a concentration of 0.997 mg/ml and HPLC was performed on YMC C18 column (5μmol/L, 250 × 4.6 mm ID) with a mobile phase consisting of methanol: water (60:40) at a flow rate 1 ml/min. Detection was by an Agilent 1260 Series HPLC with diode-array detector at 230 nm. HPLC analysis of isolated GLA confirmed its purity to 99% as shown in Figure 1B. 

### Platelet isolation and aggregation

Human venous blood was obtained from healthy donors and anticoagulated 1:5 with ACD (65 mM Na3 citrate, 70 mM citric acid, 100 mM dextrose, pH 4.4). Platelet-rich plasma (PRP) was obtained by centrifuging at 900 rpm for 20 minutes[18]. Gel-filtered platelets were prepared as described[19]. Briefly, The Sepharose^TM^ 2B was packed in PBS in a column and PRP was applied to the column. Platelets were eluted using Tyrode’s buffer to a series of 1.5 mL tubes. The collected platelets in each tubes were counted, combined, and adjusted to 2.5 × 10^8^/mL using Tyrode’s buffer for experiments. Platelets aggregation was performed in a ChronoLog aggregometer (Havertown, PA). Platelets were preincubated with vehicle or GLA for 10 min at 37°C in a cuvette. Before adding agonists, CaCl_2_ (1mM) and fibrinogen (200μg/ml) were added. Aggregation assay was started with 0% aggregation baseline and then an agonist was added to observe the percentage of platelet aggregation with stirring at 900 rpm.

### Platelet viability assay

Gel-filtered platelets (2.5×10^8^/ml) were preincubated with GLA or vehicle and then incubated with Alamar Blue reagent (Invitrogen, Carlsbad, CA, USA) for 4 hours at 37°C. The fluorescence was acquired on a SpectraMax M Series Microplate Reader (Molecular Devices, Sunnyvale, CA, USA). The excitation and emission wavelengths were 570 nm and 585 nm, respectively.

### PS Externalization Assay

As previously described[20], Gel-filtered platelets (2×10^7^/ml) were preincubated with GLA or dibucaine (500μmol/Lol/L) at room temperature for 15 min. Annexin V binding buffer (0.01M Hepes, pH 7.4, 0.14M NaCl, 2.5 mM CaCl_2_) was mixed with pre-treated platelets and annexin V-APC at a 50:50:1 ratio. Samples were gently mixed and incubated at room temperature for 15 min in the dark, then analyzed by flow cytometry.

### Immunoblotting

Platelets (2.5×10^8^/ml) pretreated with vehicle or GLA (0, 0.0025, 0.01, and 0.1μg/ml) for 10 min were stimulated with agonists at 37°C. The reaction was stopped by the addition of sodium dodecylsulfate (SDS) sample buffer (62.5 mM Tris-HCl, 2% SDS, 10% glycerol, 50 mM dithiothreitol, 0.1% bromophenol blue, pH 6.8) for dose response studies. For time course studies, platelets were pretreated with 0.1μg/ml of GLA and stimulated with agonists. The reaction was stopped at different time points. Samples were boiled for 5 min at 95°C and the proteins were separated on 10% SDS polyacrylamide gel electrophoresis (SDS-PAGE) gel. The proteins were then transferred to a nitrocellulose membrane and probed for Syk and phospho-Syk, LAT and phospho-LAT, and PLCγ2 and phospho-PLCγ2. After incubation with the goat anti-rabbit IRDye 800CW or goat anti-mouse IRDye 800CW (LI-COR Biosciences, Lincoln, NE, USA), densitometric band scanning was performed using an Odyssey Infrared Imaging System (LI-COR Biosciences).

### Platelet adhesion on collagen-coated surface under flow Conditions

The human whole blood was used for platelet adhesion assay under flow. The experiments were performed using BioFlux^TM^ 200 setup (Fluxion Biosciences, USA) following manufacture’s instruction. Briefly, the channels were primed and coated with collagen I (200μg /ml) for 1 hour at room temperature and then blocked with PBS containing 0.5% BSA for 1 hour. The human whole blood was treated by GLA or vehicle and labeled with calcein-AM (Molecular Probes, Eugene, OR, USA) at a final concentration of 10μmol/L for 30 minutes at 37°C. Blood was perfused in the channels at 1000s^-1^ and observed under fluorescent microscope for platelet adhesion and aggregates[21]. 

### Flow cytometry

Gel-filtered platelets were preincubated with GLA and then stimulated by convulxin or thrombin. Platelets were fixed with formaldehyde and stained with PE-conjugated mouse anti-human P-selectin antibody (eBioscience, San Diego, CA，USA) and FITC-conjugated PAC-1, respectively. Fluorescence-conjugated mouse IgG was used as isotype control (Santa Cruz Biotechnology). Cells were analyzed using BD FACS Calibur Flow Cytometer (BD Biosciences, USA).

### FeCl3-induced carotid artery injury model

FeCl3-induced carotid artery injury was performed as previously described with some modifications[22,23]. Briefly, GLA (10mg/kg) or vehicle was administered intraperitoneally to mice (C57BL/6, age 6-8 weeks), and after anesthesia using 7% chloral hydrate, the left carotid artery of mice was surgically exposed by blunt dissection. A 1.0×2.0-mm filter paper soaked in 7.5% FeCl3 was applied to the surface of the adventitia of the exposed artery for two minutes. After removal of the filter paper, the artery was washed with PBS and an imaging ultrasound gel (MS400-0090; VisualSonics) was placed in the surgical wound to allow Doppler monitoring. The artery was identified using a small animal blood flow transducer (MS400, 18-38 MHz; VisualSonics) and the color Doppler mode of the VisualSonics Vevo model 2100 flowmeter. Time to occlusion of the carotid artery after the application of 7.5% FeCl3 was measured using Visual Sonics View 2100. The operator was blinded to mice that infused either GLA or vehicle while performing all experiments.

### Tail bleeding time

Tail bleeding times were determined as previously described[22]. Briefly, GLA or vehicle was administered intraperitoneally to male mice (C57BL/6, age 6-8 weeks). After 45min, mice were anesthetized with pentobarbital (100 mg/kg, i.p.) and placed prone on a heating pad from which the tail protruded. The distal 5 mm of the tail was transected and immediately immersed in 12 ml 0.9% sodium chloride for 10 min at 37°C, and the time to bleeding cessation was recorded. 

### Statistical analysis

Data were analyzed by using GraphPad Prism 5.0 software and presented as means ± standard error of the mean. The statistical significance was determined by one-way ANOVA analysis of variance with the Bonferroni post test for multiple groups. The Student t test was used to calculate P values for differences. Differences were considered significant at P<0.05.

## Results

### GLA selectively inhibits platelet aggregation induced by collagen and CRP

**Figure 2:**
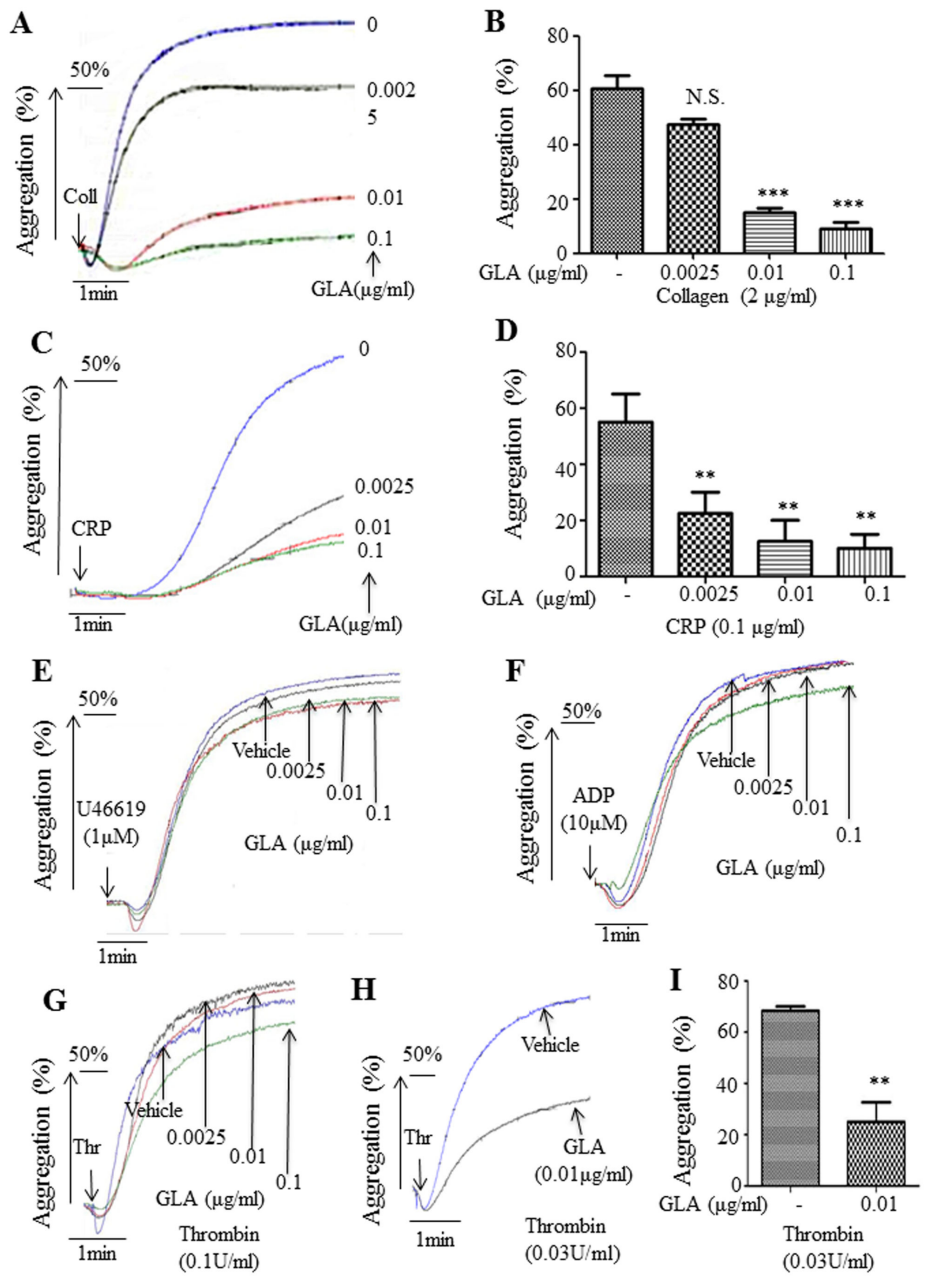
Effects of GLA on agonist-induced platelet aggregation. Gel-filtered platelets (2.5×10^8^/ml) were pre-incubated for 10 min with different concentrations of GLA (0.0025, 0.01, and 0.1μg/mL) or vehicle (DMSO). Platelet aggregation was initiated with collagen (2μg/ml) (A), CRP (0.1μg/ml) (C), U46619 (1μmol/L) (E), ADP (10μmol/L) (F), and thrombin of both a high dose (0.1U/ml) (G) and a low dose (0.03U/ml) (H). Maximum extent of platelet aggregation from three individual experiments was plotted in the bar charts (mean ± standard error, n=3). The statistical significance was determined by one-way ANOVA analysis of variance with the Bonferroni post test (B, D, and I). **P<0.01. ***P<0.001. N.S., not significant compared to vehicle control. E, F, and G are representatives of at least three individual experiments.

To investigate the effect of GLA on platelet activation, we performed platelet aggregation induced by a panel of agonists. Gel-filtered human platelets were incubated with GLA (0.0025-0.1μg/ml) or solvent alone for 10 min at 37°C and then stimulated with collagen (2μg/ml), ADP (10μmol/L), U46619 (1μmol/L), or thrombin (0.1U/ml). Results showed that GLA (0.01μg/ml, 0.1μg/ml) significantly inhibited platelet aggregation induced by collagen (P<0.001) (Figure 2A&2B) with IC50 of 0.00725μg/ml and CRP, a GPVI selective agonist (P<0.01) (Figure 2C&2D) with IC50 of 0.00062 μg/ml compared with vehicle-treated platelets. However, none of the concentrations of GLA significantly inhibits platelet aggregation induced by other agonists (Figure 2E-2G), implying a selective effect of GLA on the GPVI signaling pathway. Interestingly, GLA seemed more efficient in inhibiting CRP-induced platelet aggregation as the inhibitory concentration was as low as 0.0025μg/ml. Therefore, our data suggested that relatively lower doses of GLA selectively inhibits platelet aggregation via GPVI signaling pathway. Additionally, when we examined the effect of GLA on the low-dose thrombin (0.01, 0.03, and 0.05U/ml)-induced platelet aggregation, we found that GLA significantly inhibited platelet aggregation induced by 0.03U/ml thrombin (P<0.01) (Figure 2H&2I), but not 0.05U/ml thrombin (data not shown), implying a potentially additional target of GLA in platelet activation.

**Figure 3:**
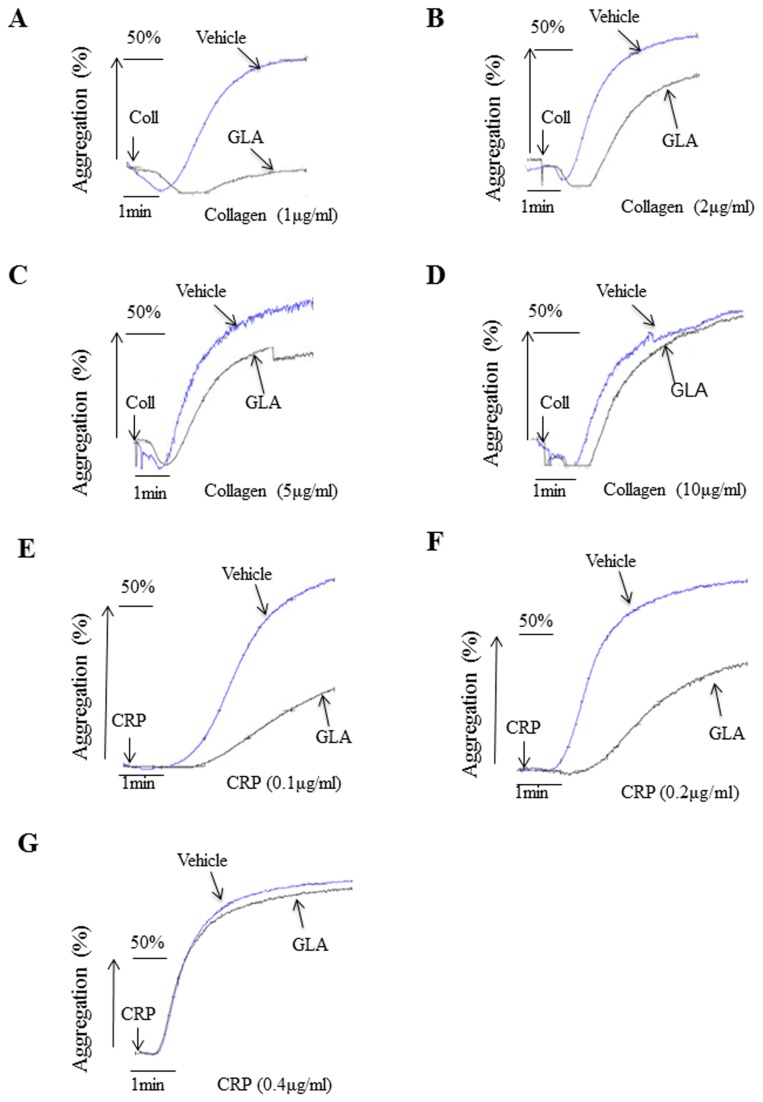
Effects of GLA on platelet aggregation induced by different concentration of collagen and CRP. Gel-filtered platelets (2.5×10^8^/ml) were pre-incubated for 10 min with GLA (0.01μg / mL) or vehicle (DMSO). Platelet aggregation was initiated with 1μg/ml (A), 2μg/ml (B), 5μg/ml (C), and 10μg/ml (D) of collagen, and 0.1μg/ml (E), 0.2μg/ml (F), and 0.4μg/ml of CRP (G). The aggregation curves are the representatives of at least three individual experiments.

Using the effective inhibition dose of GLA (0.01μg/ml), we tested whether GLA inhibits platelet aggregation induced by higher doses of collagen. Results showed that the GLA (0.01μg/ml) inhibits collagen-induced platelet aggregation within the concentrations of 1- 5μg/ml (Figure 3A-3D). We also measured the inhibition of GLA on different concentrations of CRP-induced platelet activation. We showed that GLA (0.01μg/ml) inhibited CRP-induced platelet aggregation within the concentrations of 0.1- 0.2μg/ml (Figure 3E-3G).

### GLA Did Not Induce the Toxic or Apoptotic Effects of Platelets

**Figure 4:**
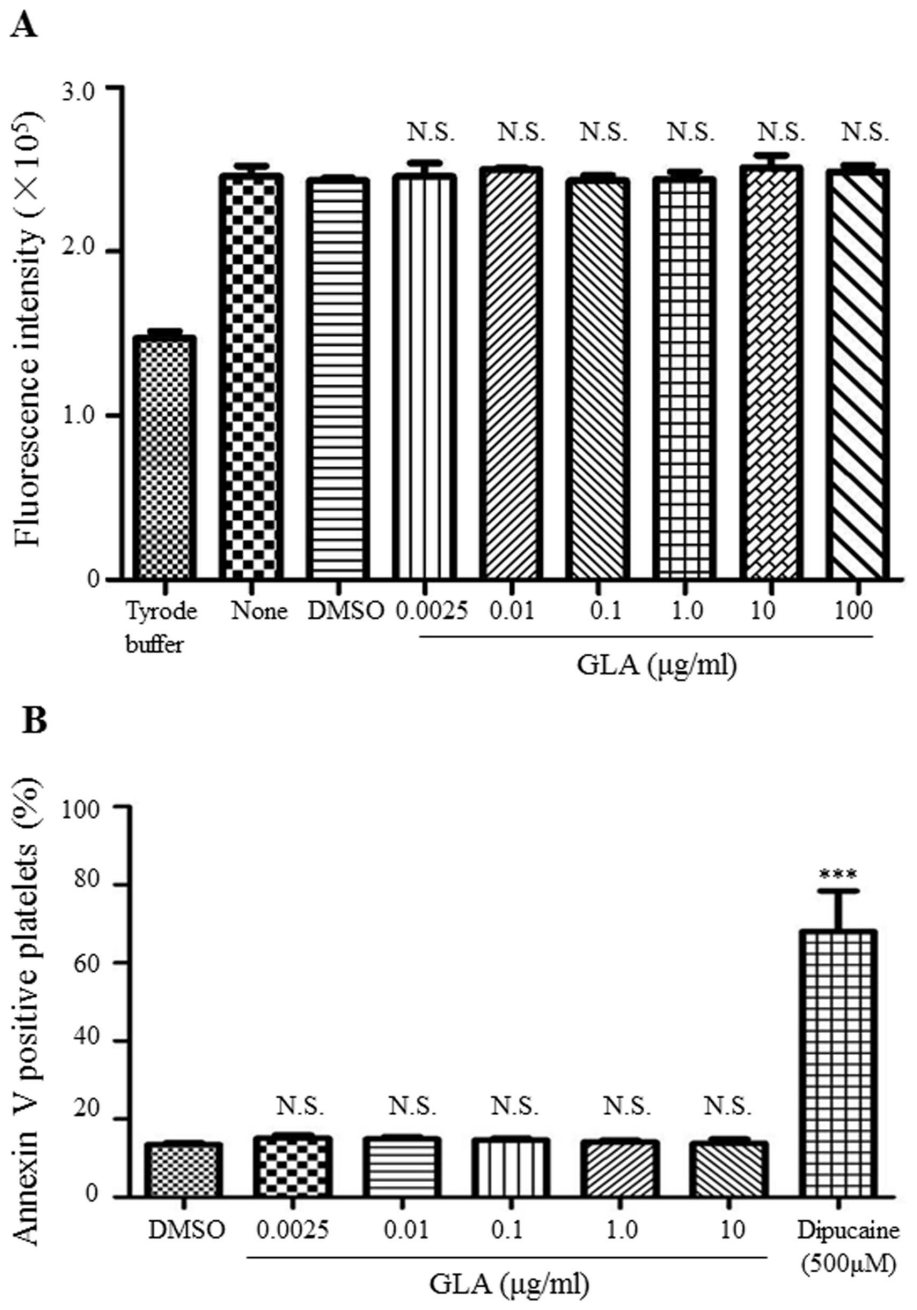
Evaluation of the potential toxic and apoptotic effect of GLA on platelets. (A) In platelet viability assay for the toxic effect of GLA, gel-filtered platelets (2.5×10^8^/ml) were preincubated with GLA (0.0025-100μg/ml) or vehicle and then incubated with Alamar Blue reagent for 4 hours at 37°C. The fluorescence was acquired with the excitation and emission wavelengths of 570 nm and 585 nm, respectively, and plotted in the bar charts (mean ± standard error, n=3). (B) In PS externalization assay, gel-filtered platelets (2×10^7^/ml) were preincubated with GLA (0.0025-10μg/ml) or vehicle or dibucaine (500μmol/Lol/L) at 37°C for 15 min, and then mixed with Annexin V binding buffer and Annexin V-APC. Samples were analyzed by flow cytometry and the percentage of annexin V positive platelets was calculated (mean ± standard error, n=3). ***P<0.001. N.S., not significant compared to vehicle control. The statistical significance was determined by one-way ANOVA analysis of variance with the Bonferroni post test.

To examine whether the inhibition effect of GLA on agonist-induced platelet aggregation is due to its potential toxic or apoptotic effect, we performed Alamar Blue staining and platelet PS exposure assay. Results showed that treatment of platelets with GLA (0, 0.0025, 0.01, 0.1, 1.0, 10μg/ml) for 15min at 37°C did not cause any significant toxic or apoptotic effect on platelets, confirming the inhibitory effect of GLA on platelet aggregation (Figure 4A&4B). 

### GLA inhibits tyrosine phosphorylation of Syk, LAT, and PLCγ2 in the GPVI signaling pathway

**Figure 5:**
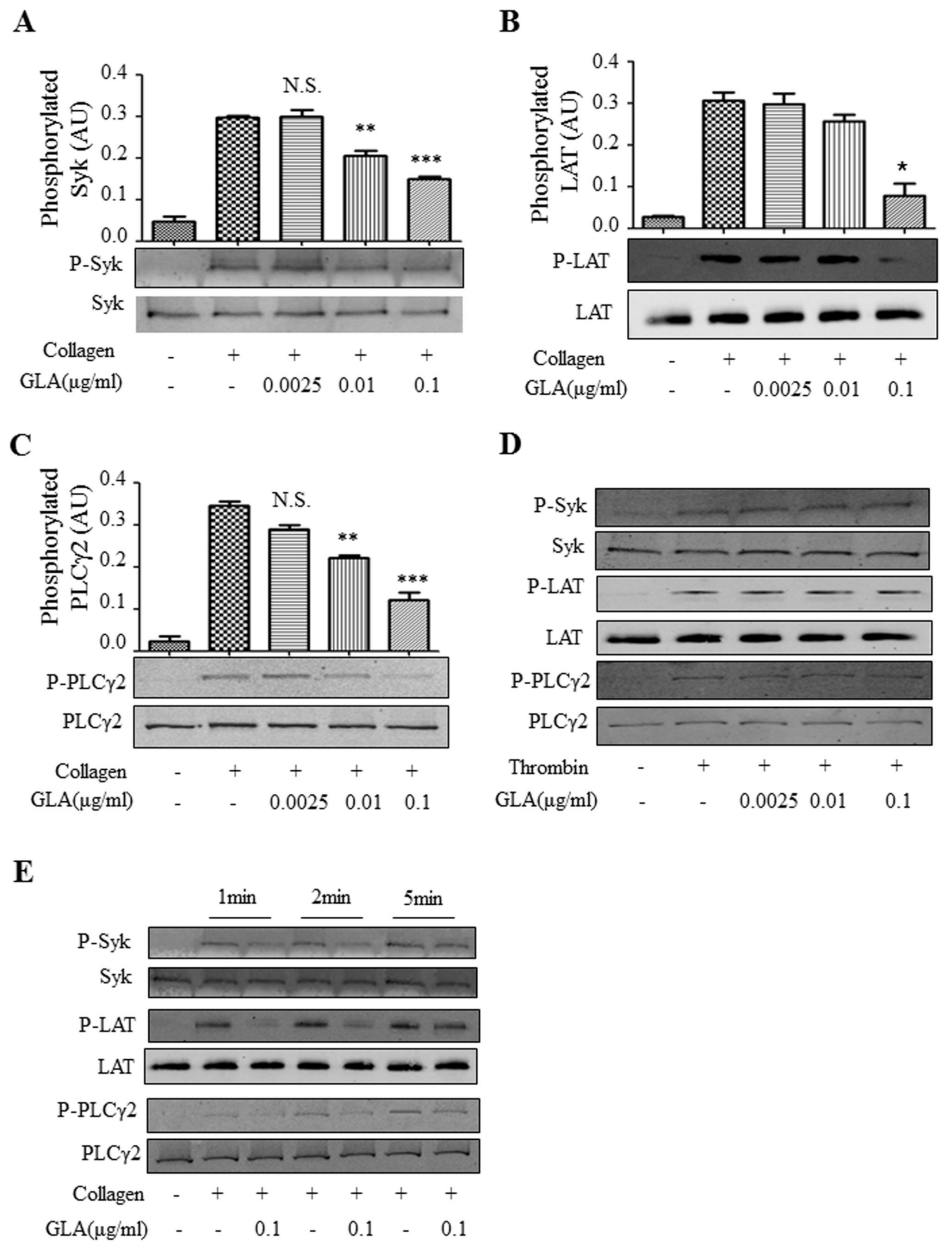
Influences of GLA on platelet intracellular signaling. Gel-filtered platelets (2.5×10^8^/ml) were pre-incubated with vehicle (DMSO) or GLA (0.0025, 0.01, 0.1μg/ml), and stimulated with collagen (2μg/ml) with stirring at 900 rpm in an aggregometer at 37°C for 60 seconds. Platelets were lysed, and immunoblotted using the corresponding antibodies recognizing total and phosphorylated Syk (A), total and phosphorylated LAT (B), and total and phosphorylated PLCγ2 (C). After incubation with the goat anti-rabbit IRDye 800CW or goat anti-mouse IRDye 800CW (LI-COR Biosciences, Lincoln, NE, USA), densitometric band scanning was performed using an Odyssey Infrared Imaging System (LI-COR Biosciences) (mean ± standard error, n=3). **P <0.01. ***P <0.001. N.S., not significant compared to vehicle control. The statistical significance was determined by one-way ANOVA analysis of variance with the Bonferroni post test. Same methods were used to measure the time course of GLA inhibition (D) and thrombin control (E) for the phosphorylation of the three signaling molecules in the GPVI pathway. The gels are the representatives of at least three individual experiments.

Given the fact that lower concentration of GLA inhibits platelet aggregation induced by collagen and CRP, we next examined the effect of GLA on collagen-stimulated signal transduction downstream of GPVI signaling pathway. Activation of GPVI collagen receptor results in tyrosine phosphorylation of the associated receptor-complex protein, the FcR γ-chain mediated by the Src-family kinases Fyn and Lyn. This leads to the recruitment and activation of the cytosolic tyrosine kinase Syk. The effect of GLA on the level of tyrosine phosphorylation of Syk following stimulation with collagen was therefore examined. Results showed that GLA decreased the level of collagen-stimulated tyrosine phosphorylation of the Syk in a concentration-dependent manner (Figure 5A). Activation of Syk following its association with the activated receptor complex including LAT results in tyrosine phosphorylation and recruitment of phospholipase Cγ2 (PLCγ2) [24]. We therefore also investigated the effect of GLA on collagen-stimulated tyrosine phosphorylation of LAT and PLCγ2. Results showed that the levels of tyrosine phosphorylation of LAT and PLCγ2 were also inhibited in a concentration-dependent manner by GLA (Figure 5B & 5C). Time course studies showed that inhibition of GLA on collagen-induced tyrosine phosphorylation of Syk, LAT, and PLCγ2 occurred at 1-2min of platelet activation (Figure 5D). On the contrary, GLA did not inhibit thrombin (0.1U/ml)-induced tyrosine phosphorylation of Syk, LAT, and PLCγ2 (Figure 5E) 

### GLA inhibits platelet granule secretion and inside-out activation of the integrin αIIbβ3

**Figure 6:**
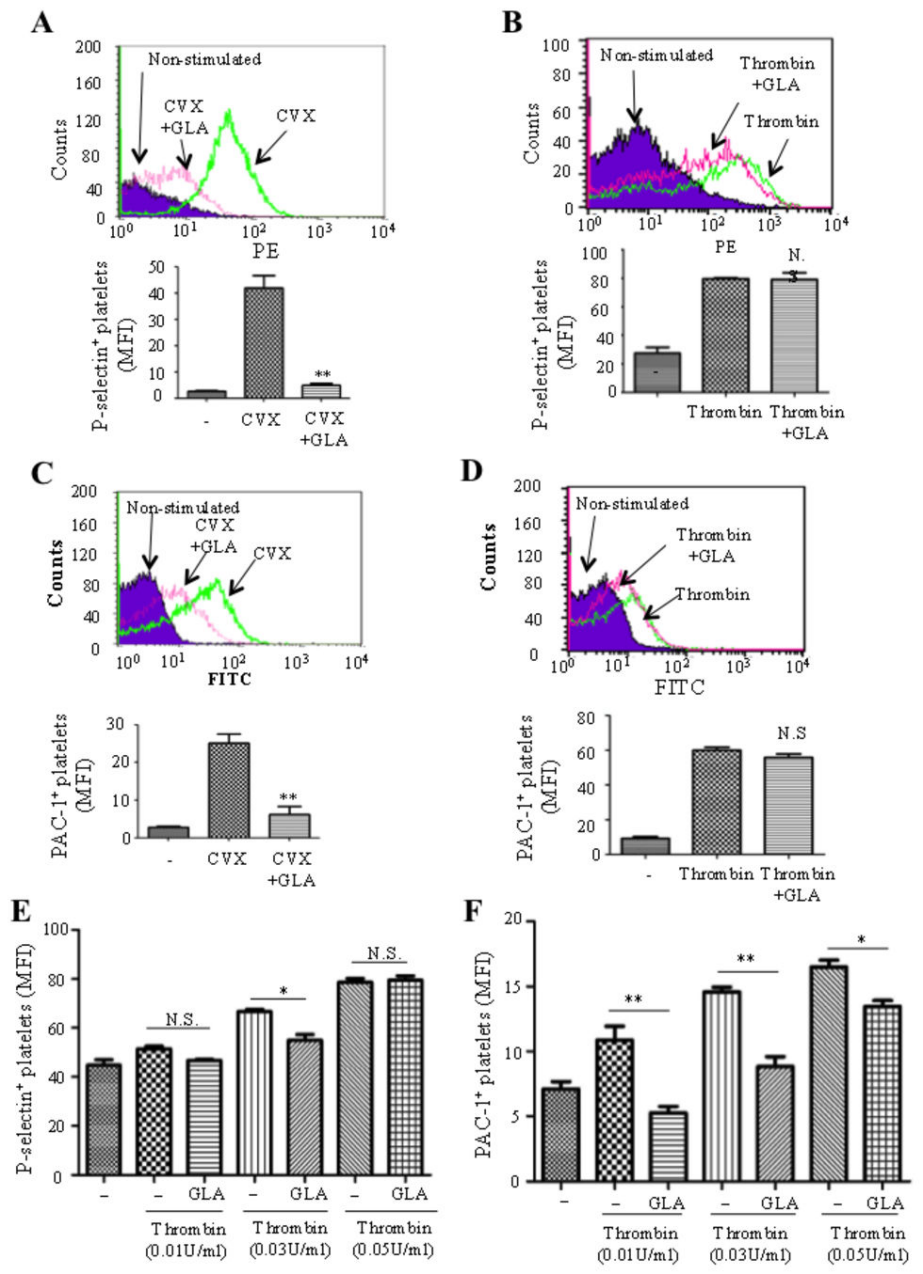
Effects of GLA on single-platelet activation. Gel-filtered platelets (2.5×10^8^/ml) were pre-incubated with vehicle (DMSO) or GLA (0.5 μg/ml) for 10 min in the presence of PE-conjugated P-selectin antibody or FITC-conjugated PAC-1. Samples were then challenged with CVX (0.2nM) (A, C) or thrombin (0.1U/ml) (B, D) and incubated for 10 min. P-selectin expression (A, B) and PAC-1 binding (C, D) were monitored by flow cytometry. Similarly, using the effective inhibitory dose of GLA (0.01μg/ml), P-selectin secretion (E) and integrin activation (F) induced by lower doses of thrombin were tested. Data plotted are means ± standard errors of three individual experiments. *P < 0.05. **P < 0.01. N.S., not significant compared to vehicle control.. The statistical significance was determined by one-way ANOVA analysis of variance with the Bonferroni post test.

As the activation of GPVI and its interaction with the downstream molecules leads to platelet P-selectin activation and the inside-out signaling of the integrin αIIbβ3, we examined the influence of GLA on those events. Human platelets were pre-incubated with GLA (0.5μg/ml) in the presence of fluorescent PE-conjugated P-selectin antibody and then challenged with convulxin (0.2nM), a selective glycoprotein VI ligand, for 20 minutes. P-selectin expression of platelets was monitored by flow cytometry. As shown in Figure 6A, convulxin increased P-selectin expression, but the increase was reduced in the presence of GLA (p<0.01). However, we did not found any inhibition of platelet granule secretion when thrombin was used to stimulate platelets (Figure 6B). To test whether GLA inhibits platelet inside-out signaling of the integrin αIIbβ3, PAC-1 was used as it recognizes an epitope on the integrin αⅡbβ3 complex of activated platelets at or near the platelet fibrinogen receptor[24]. Similarly, human platelets were pre-incubated with GLA (0.5μg/ml) in the presence of FITC-conjugated PAC-1 and then challenged with convulxin (0.2nM) for 20 minutes. PAC-1 binding of single platelet was monitored by flow cytometry. As shown in Figure 6C, convulxin increased platelet PAC-1 binding and the increase was reduced in the presence of GLA (p<0.01). Again, we did not find any inhibition of platelet granule secretion when thrombin was used to stimulate platelets (Figure 6D).

Since we found GLA inhibits low-dose thrombin-induced platelet aggregation, we further titrated the thrombin concentrations that were inhibited by GLA for its ability to induce platelet aggregation using flow cytometric analysis of platelet granule secretion and integrin activation. As shown in Figure 6E and 6F, GLA only significantly inhibited 0.03U/ml thrombin-induced P-selectin secretion (P<0.05) consistent to the aggregation assay (Figure 2H&2I). However, GLA was found to significantly inhibit all three concentrations of thrombin (0.01, 0.03, and 0.05U/ml)-induced integrin activation tested (P<0.01, P<0.01, and P<0.05, respectively). 

### Effects of GLA on platelet function ex vivo

**Figure 7:**
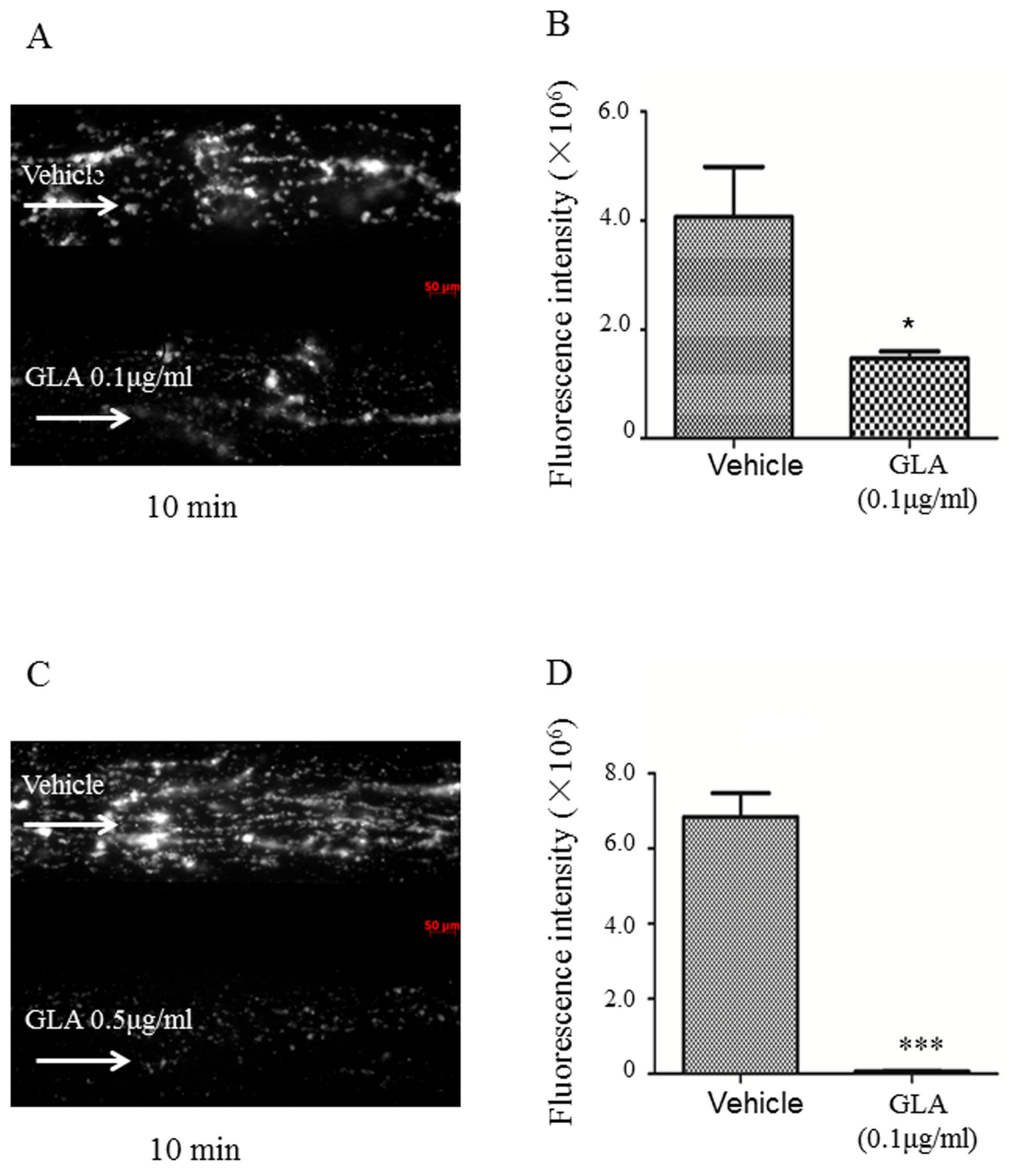
Effects of GLA on platelet adhesion on collagen-coated surfaces. **H**uman whole blood was labeled with Calcein-AM, treated with (lower channels) or without (upper channels) 0.1μg/ml (A) or 0.5μg/ml (B) of GLA, and perfused over collagen-coated surface for 30 min at a shear rate of 40 dyn/cm^2^ (1000s^-1^). Platelet adhesion and aggregates were observed under a fluorescence microscope. Photos were taken at 10min (A) after flow. Arrow indicates the flow direction. Data plotted are means ± standard errors of fluorescence intensity of three individual experiments (*P < 0.05, ***P < 0.01). The Student t test was used to calculate P values for differences.

Functional GPVI plays an important role in platelet adhesion to collagen. We next examined the effect of GLP on platelet adhesion on collagen-coated surface under flow using a microfluidics flow chamber. We perfused anticoagulated blood that has been labeled by calcein-AM and preincubated with GLA over a collagen-coated surface in the microfluidics flow chamber (1000s^-1^)[25] for 10min. We observed that an initial monolayer of platelets adhered to the collagen, after which additional platelets accumulated on those that arrived first, forming platelet aggregates. The fluorescence intensity from platelets on the collagen surface was calculated. Data analysis showed that the extent of platelet accumulation were reduced by over 60% in the presence of 0.1μg/ml of GLA (P<0.05) and almost completely wiped out in the presence of 0.5μg/ml GLA (P<0.001) at 10min after perfusion, indicating that GLA attenuates platelet adhesion and aggregation on collagen surface (Figure 7A-7D). 

### GLA inhibited thrombus formation in vivo, but did not cause bleeding tendency in low dose GLA-treated animals

**Figure 8:**
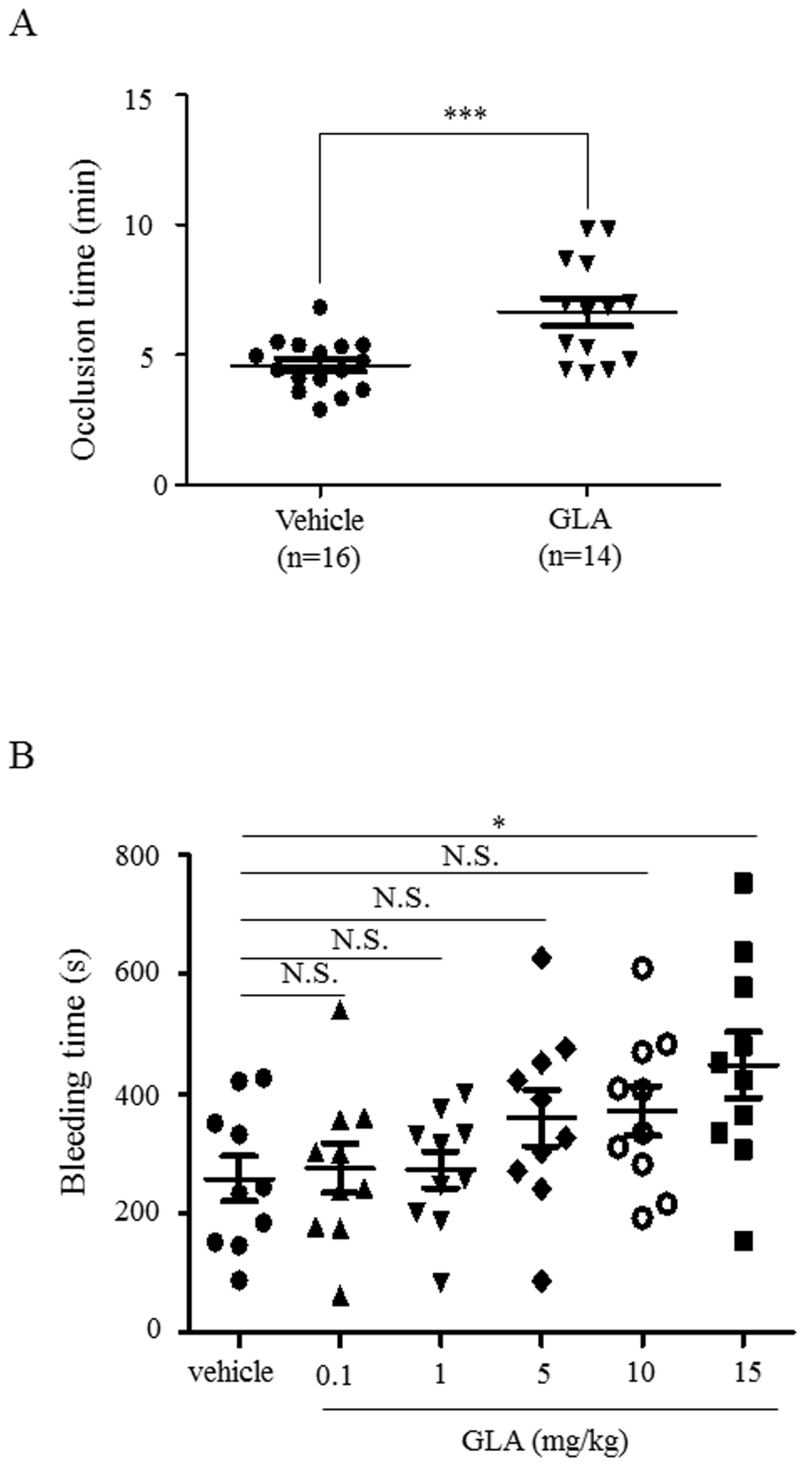
GLA inhibits thrombus formation in vivo, but did not extend the tail bleeding time in mice. GLA was given at a single bolus i.p injection with solvent as a vehicle control. (A) FeCl3-induced carotid artery injury mouse model was performed and the time to occlusion was measured using Visual Sonics View 2100 and analysed using Student T test (mean ± standard error, P < 0.001). (B) The distal 5 mm of the tail of anesthetized mice was transected and the time to bleeding cessation was recorded. Data were analyzed using GraphPad Prism 5.0 software with the Bonferroni post test for multiple groups (mean ± standard error, n=10 for each group) *P < 0.05. N.S., not significant.

To study the effect of GLA on thrombus formation in vivo, FeCl3-induced carotid artery injury mouse model was performed and the time to FeCl3-induced carotid artery occlusion was measured. Mice were randomly grouped into two groups and injected with either GLA (10 mg/kg) or solvent as a vehicle control at a single bolus via i.p. injection. Although there was overlap in some of the individual results, the average time to occlusion increased by 30.3%, from 4.6 ± 0.25min in the vehicles (n=16) to 6.6 ± 0.52min in the GLA group (n=14) (mean ± error. P < 0.001) (Figure 8A). To evaluate the bleeding risk of using GLA, the effect of GLA administration on the tail bleeding time was examined. Mice were randomly grouped into 6 groups (10 mice in each group) and GLA (vehicle, 0.1, 1.0, 5.0, 10, 15 mg/kg) was administered for each group. Forty-five minutes after GLA administration, the first bleeding time of tail snip was recorded. Results showed that administration of lower doses of GLA (0.1, 1.0, 5.0, 10 mg/kg) did not extend bleeding time as compared to the vehicle group. However, higher dose of GLA (15 mg/kg) caused a significant extension of bleeding time compared to the vehicle control (P>0.05). Therefore, our data clearly indicate that GLA has no bleeding risk at the dose (<10mg/kg) that we used to inhibit thrombus formation in carotid artery injury model (Figure 8B), but attention for the bleeding risk has to be given when a higher dose of GLA is used. 

## Discussion

To identify potential compounds from natural products that have antiplatelet and antithrombotic effect without bleeding tendency as our systemic drug discovery project, we have recently isolated and purified *ent*−kauranoid diterpenoids, glaucocalyxin (GLA), glaucocalyxin B (GLB), and glaucocalyxin C (GLC) from *Rabdosia japonica* var. *galucocalyx*. Both GLA and GLB have a αβ-unsaturated ketone at C-15, C-16, and C-17, while GLC has a α-orientated hydroxyl group at C-15 and a methylene group at C-16 and C-17. The structural difference between GLA and GLB is that the hydroxyl group at C-14 of GLA has been acetylated in GLB. Since all three diterpenoid compounds inhibit platelet aggregation (data not shown), we focused on GLA in this study and investigated its effect on platelet activation and thrombus formation as well as the underlying molecular mechanism. We found that GLA inhibits platelet aggregation in response to collagen through the inhibition of GPVI-mediated signaling in vitro in a concentration-dependent manner. It also inhibited platelet activation induced by low dose of thrombin. Platelet secretion, integrin activation, and platelet adhesion on a collagen surface were also inhibited by GLA. In vivo studies showed that GLA decreases the thrombus formation without bleeding tendency in relatively low concentrations. 

Previous reports[16,17] showed that GLA (1-100μmol/L) inhibits rabbit platelet aggregation induced by ADP, AA, and PAF. Consistently, preincubation of human platelets with relatively higher concentrations of GLA (5-50 μg/ ml) significantly inhibited platelet aggregation in response to most of the platelet agonists including collagen, thrombin, ADP, and U46619 (data not shown). However, the inhibitory effect of GLA on collagen-stimulated platelet aggregation was notably potent in comparison with other agonists as the inhibition even occurs at as low as 0.01μg/ml (0.03μmol/L) of GLA, at which other agonist-induced platelet aggregation was not inhibited. The effect of high dose of GLA on platelet aggregation could potentially be due to the effect of GLA on multiple molecules in platelets as GLA was shown to inhibit the PAF biosynthesis, decrease TXA level, and increase the levels of PGE2 and cAMP in high doses[16,17,26]. It could be possible that high dose of GLA may induce the toxicity and the apoptosis of platelets. However, the viability assay and PS externalization assay excluded this possibility (Figure 4).

The selective inhibitory effect of low dose GLA on collagen-induced platelet aggregation led us to ask whether GLA inhibits platelet aggregation via GPVI pathway. Platelets are known to have two major receptors for collagen, the integrin α2β1 and the glycoprotein VI/FcRc-chain complex (GPVI), as well as a number of minor receptors of uncertain significance[27]. We used collagen-related peptide (CRP), a GPVI specific agonist, to define the characteristics of the GPVI-mediated platelet activation. As expected, our results showed that GLA inhibits CRP-induced platelet aggregation in a dose-dependent manner, confirming that GLA inhibits platelet activation via GPVI signaling pathway. However, GLA may affect platelet activation through GPCR as well because GLA inhibits platelet aggregation induced by lower dose of thrombin (0.03U/ml) although GLA does not inhibit high dose thrombin (0.1 U/ml)-induced platelet aggregation. How GLA affects low dose thrombin-induced platelet activation and what is the integral effect of GLA on both GPVI pathway and GPCR need to be further studied.

To study the molecular mechanism and the consequence of GLA on platelet activation in vitro and ex vivo, we examined the effect of GLA on the downstream signaling of GPVI pathway, platelet secretion, the inside-out activation of integrin αIIbβ3, and platelet adhesion on collagen-coated surface. As expected, incubation of GLA reduced collagen-induced phosphorylation of three major molecules, Syk, LAT, and PLCγ2 in GPVI signaling pathway. However, the exact target(s) of GLA in platelets is unknown. The influence of GLA on single platelet activation was further investigated by two separate assays and we showed that GLA inhibits p-selectin expression and αIIbβ3 activation induced by collagen and lower doses of thrombin. Microfluidic chamber assay is a recently developed design to study platelet adhesion on the coated surface under flow condition[25]. GLA-treated platelets showed less accumulation than the untreated platelets in a dose-dependent manner. 

For in vivo studies, we examined the effect of GLA on thrombus formation using FeCl3-induced carotid artery injury model, which measures the thrombosis in the medium or large artery, and showed that GLA negatively regulates thrombus formation. Moreover, the thrombi in GLA-treated mice seemed less stable as there were 8 reflows and 2 non-occlusions in 16 mice tested compared to the vehicle control group with 4 reflows out of 16 mice (data not shown), implying that GLA possibly affects the stability of the thrombi. The benefit of an antithrombotic agent is the balance between efficacy in reduction of symptomatic thrombotic events and the risk for hemorrhage. Although GLA showed antiplatelet and antithrombotic effect, it has to be safe to be developed as a preventive or therapeutic agent. Our study confirmed that relatively lower doses (0.1-10mg/kg) of GLA administered via intraperitoneal injection did not cause significant bleeding tendency. However, a higher dose of GLA (15mg/kg) caused a significant increase in bleeding time. It should be pointed out that the doses used *in vitro* and *in vivo* in this study are not parallel since we did not measure the dynamics of GLA in plasma after injection. Therefore, it is unknown whether the dose used here *in vivo* represent the low dose response *in vitro*. Further study is required to evaluate the effective and selective doses of GLA on antiplatelet and antithrombotic effect without bleeding tendency. 

In summary, we have isolated and purified GLA, and established that the potent inhibitory action of GLA on platelet activation and thrombus formation is preferentially via GPVI-mediated signaling pathway. Although further work is required to determine the targets of GLA in platelets, the observations described in this study supply evidence that this natural compound may become a potential antiplatelet and antithrombotic agent. 
